# Noninvasive genetic sampling reveals intrasex territoriality in wolverines

**DOI:** 10.1002/ece3.1983

**Published:** 2016-02-09

**Authors:** Richard Bischof, Espen R. Gregersen, Henrik Brøseth, Hans Ellegren, Øystein Flagstad

**Affiliations:** ^1^ Department of Ecology and Natural Resource ManagementNorwegian University of Life SciencesÅsNorway; ^2^ Norwegian Institute for Nature ResearchTrondheimNorway; ^3^ Department of Evolutionary BiologyUppsala UniversityUppsalaSweden

**Keywords:** Animal movements, home range overlap, kernel home range, large carnivores, resource selection function, territory takeover

## Abstract

Due to its conspicuous manifestations and its capacity to shape the configuration and dynamics of wild populations, territorial behavior has long intrigued ecologists. Territoriality and other animal interactions in situ have traditionally been studied via direct observations and telemetry. Here, we explore whether noninvasive genetic sampling, which is increasingly supplementing traditional field methods in ecological research, can reveal territorial behavior in an elusive carnivore, the wolverine (*Gulo gulo*). Using the locations of genotyped wolverine scat samples collected annually over a period of 12 years in central Norway, we test three predictions: (1) male home ranges constructed from noninvasive genetic sampling data are larger than those of females, (2) individuals avoid areas used by other conspecifics of the same sex (intrasexual territoriality), and (3) avoidance of same‐sex territories diminishes or disappears after the territory owner's death. Each of these predictions is substantiated by our results: sex‐specific differences in home range size and intrasexual territoriality in wolverine are patently reflected in the spatial and temporal configuration of noninvasively collected genetic samples. Our study confirms that wildlife monitoring programs can utilize the spatial information in noninvasive genetic sampling data to detect and quantify home ranges and social organization.

## Introduction

Territoriality is a ubiquitous behavioral phenomenon that transcends taxonomic phyla and comes in many shapes and forms (Potts and Lewis 2014). Regardless of how it is manifested, territoriality is thought to enhance the fitness of individuals or groups by securing exclusive or privileged access to resources (Brown and Orians 1970). Emerging from behavior at the individual or group level, territoriality shapes the spatial arrangement and dynamics of entire populations (Lomnicki 1983; Adams 2001) and can therefore have significant implications for management and conservation (Verdade 1996; Hoffman and O'Riain 2012; Eads et al. 2014).

When studying territoriality in situ, ecologists rely mainly on telemetry or direct observations (Doncaster and Macdonald 1991; Minta 1993; Katnik et al. 1994; Boydston et al. 2001; Gese 2001). For many other fields of inquiry, these traditional direct methods are increasingly supplemented with and in some cases replaced by noninvasive genetic sampling, that is, the collection of hair, feathers, scat, and other material left behind by animals, followed by DNA extraction and genetic analysis. Noninvasive genetic sampling is being applied to the study of many natural phenomena associated with wild populations and communities, including abundance, population dynamics, species diversity, foraging behavior, and population genetics (Taberlet et al. 1999; Mills et al. 2000). Territoriality leads to conspicuous space‐use patterns, and one would expect this to be reflected in the spatiotemporal configuration of genetic samples. The potential use of noninvasive genetic sampling to explore spatial behavior was pointed out early on (Kohn and Wayne 1997; Kohn et al. 1999), yet, surprisingly few studies have constructed home ranges or territories from the locations of genetic samples (Davoli et al. 2013; Caniglia et al. 2014; to some degree Taberlet et al. 1997). We are not aware of any studies attempting to quantify territoriality/territorial exclusion based solely on the spatial information contained in genetic sampling data.

**Figure 1:**
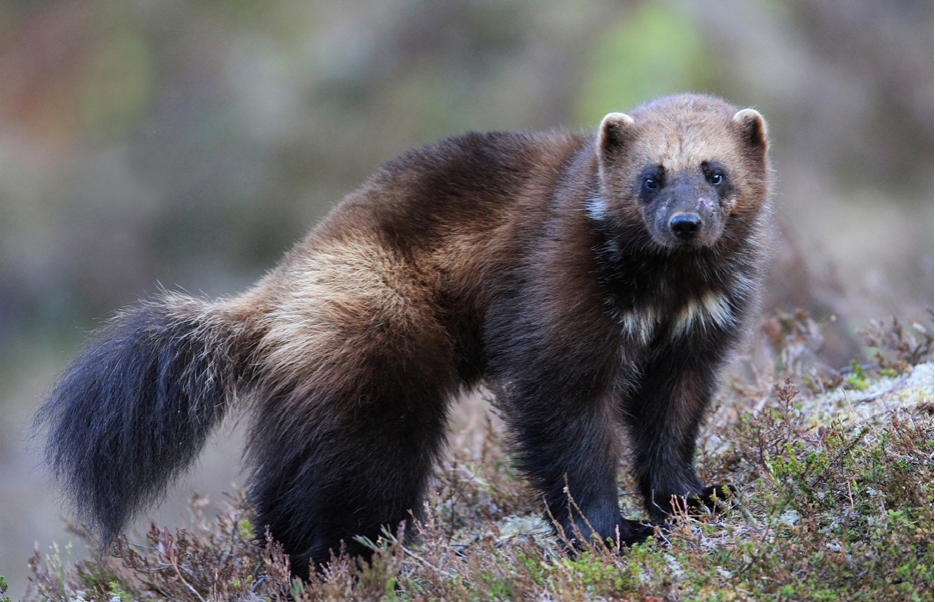
Wolverine *Gulo gulo*. Photo: Kjetil Schjølberg, Rovdata.

In this study, we test whether noninvasive genetic sampling data can reveal patterns consistent with territoriality in the wolverine (*Gulo gulo*, Fig. 1). Even among carnivores, wolverines are notorious for their elusiveness and have a reputation for being particularly difficult to study (Persson et al. 2010). Genetic sampling offers a viable alternative to traditional, invasive methods, and scat‐based sampling has become a mainstay of wolverine population monitoring in Norway (Flagstad et al. 2004; Hedmark et al. 2004; Hedmark and Ellegren 2007). Today, wolverine genetic sampling data in Scandinavia are being used primarily to estimate population dynamic parameters and abundance (Brøseth et al. 2010). However, these data also offer a thus far unexplored opportunity for testing hypotheses about spacing behavior.

There is general agreement that wolverine social structure is defined by varying degrees of intrasexual territoriality and intersexual tolerance, both in the Eurasian and North American parts of the species’ range (Banci 1994; Persson et al. 2010; Inman et al. 2012; but see Hornocker and Hash 1981). Based on published information about wolverine spatial behavior, we can construct a series of predictions to be tested using noninvasive genetic sampling relocation data. First, as is the case for other large solitary carnivore species, home ranges of male wolverines tend to be substantially larger than those of females (Landa et al. 1998; Persson et al. 2010; Inman et al. 2012). We therefore predict that individual home ranges constructed from the locations of genotyped scats are larger for males than females (P1). Second, male and female wolverines exclude individuals of the same sex to varying degrees, whereas male home ranges can overlap multiple female home ranges (Persson et al. 2010; Inman et al. 2012). We thus predict that scats of one individual are less likely to be located within an area used by another animal in its neighborhood if both animals are of the same sex (P2). Finally, no territory is held by one individual or group indefinitely; the death of a territory's owner temporarily ends exclusion and is one of the mechanisms that facilitates turnover (Beletsky 1992), presumably also in wolverines (Vangen et al. 2001). We predict that avoidance of areas used by another individual of the same sex diminishes or disappears at least temporarily following that animal's death (P3). Using an extensive individual‐based genetic data set collected during the national wolverine monitoring program in Norway, we test predictions P1–P3 and show compelling evidence that the spatial information contained in noninvasive genetic sampling data can indeed be used to detect and quantify territorial behavior in wildlife.

## Materials and Methods

### Scat collection and study area

Wolverine fecal and hair samples collected by the Norwegian Nature Inspectorate in two counties (Hedmark and Oppland) in south‐central Norway formed the basis of this study. Samples were collected annually between 2001 and 2012 as part of the national monitoring program for the species in Norway. Collections were conducted via snow tracking between October and June the following year, with a peak collection period between February and May (93.5% of samples). GPS position and date of the sampling event were recorded for each fecal sample. The study area (62°N 10°E, ~35,000 km^2^) is located in the southern part of the wolverine's distribution range in Norway, encompassing remote mountains in the west to more accessible forests in the east. Detailed descriptions of sample collection and the study area are provided elsewhere (May et al. 2006, 2008; Brøseth et al. 2010). In addition to scat‐based monitoring, we sampled muscle tissue from the 336 wolverines legally shot in Hedmark and Oppland counties and their neighboring counties during the same time period and matched their identity with those provided by noninvasive genetic samples. All wolverines legally killed in Norway are registered by the authorities, from which we obtained the location and date of death for these individuals.

### Genetic analysis

We extracted and amplified DNA material from all collected scat and tissue samples. Over the years, DNA extraction and microsatellite genotyping protocols have been modified; from manual to automated DNA extraction and by replacing singleplex PCR amplification with multiplex PCR. Earlier protocols are described in Flagstad et al. (2004) and Brøseth et al. (2010). In the most updated protocol for automated DNA extraction, we applied a Genemole DNA extraction robot, following the protocol for tissue samples provided by the manufacturer (Mole Genetics, Lysaker, Norway). Microsatellite genotyping to identify individual wolverines included eleven autosomal loci, distributed in two multiplex panels. Assuming a panmictic population across the study area, the probability of identity (pID; Waits et al. 2001) was 2.9 × 10^−7^ for unrelated wolverines, and 9.2 × 10^−4^ for siblings.

PCR amplifications for autosomal microsatellite loci were performed in 10 μL reactions containing 3.0 mmol L^−1^ MgCl_2_, 0.2 mmol L^−1^ of each dNTP, 2.0–8.0 pmol of each primer, 0.5 μg of bovine serum albumine, 0.9 units of HotStar DNA polymerase (Qiagen, Hilden, Germany), and 2 μL of undiluted DNA extract. We used a touchdown PCR program with an initial denaturation step of 95°C for 15 min. Six touchdown cycles with 94°C for 30 sec, 58°C for 30 sec decreasing 1°C each cycle, and 72°C for 1 min was followed by 26 or 33 cycles (DNA extracted from tissue and scat, respectively) of 94°C for 30 sec, 52°C for 30 sec and 72°C for 1 min, and a final extension at 72°C for 10 min. For sex determination, two Y‐chromosome‐specific markers that had been validated by scat sampling from radio‐marked individuals of known sex were used (DBY3Ggu, DBY7Ggu; Hedmark et al. 2004).

PCR products were visualized on an ABI 377 instrument (2000–2001; Applied Biosystems, Foster City, CA), a MegaBACE genetic analyzer (2002–2006; GMI Inc, Ramsey, MN), or an ABI 3730 XL DNA analyzer (2007; Applied Biosystems). The subsequent determination of allele lengths was performed with Genescan and Genotyper (2001), Genetic Profiler (2002–2006), and GeneMapper (2007‐2012). Calibration of allele lengths from the three different instruments were routinely performed using a set of reference samples run on all three instruments.

Genotyping errors caused by amplification of poor quality DNA from scat samples such as allelic dropout and false alleles can severely bias estimates of population parameters by creating “false” individuals (Mills et al. 2000; Waits and Leberg 2000). Therefore, we performed a number of control measures to ensure the quality of our genetic data. All scat samples were amplified at least three times for each microsatellite marker (the multitube approach; Taberlet et al. 1996). A single‐locus genotype was not accepted before our replicates resulted in at least three identical homozygote profiles or two identical heterozygote profiles. These criteria were based on a pilot study, where we compared genotypes obtained from scats versus blood or tissue samples from the same individuals (Hedmark et al. 2004). More than 200 single‐locus genotypes were tested, and three replicates were always sufficient for deriving the correct genotype. As an additional quality control, we calculated the quality index (QI) for individuals that were only represented with one single sample (Miquel et al. 2006). The authors of that study recommended discarding samples with QI < 0.625, but we applied even stricter criteria by elevating this threshold to 0.8, ensuring that the final data set contained reliable genotypes.

### Data analysis

#### P1: home range size and sex of wolverines

We selected individuals with at least 8 noninvasive genetic sampling relocations and calculated 95% kernel home range sizes using functions kernelUD and kernel.area in R package adehabitatHR (Calenge 2006). The threshold for the number of relocations for kernel construction was selected in order to balance the number of individuals retained for the analysis with the need for sufficient relocations for individual home range estimation. We used simple linear regression with log‐transformed home range size as the response and sex and the number of years from which noninvasive genetic sampling relocations for a given individual were available as predictor variables. The number of years was included in order to control for the fact that for most individuals, due to low annual sample sizes, home ranges were constructed over multiple years. Multiyear home ranges are likely larger than annual home ranges, even if interannual shifts in relocation clusters are small. The effect of number of years with detections on home range size was modeled using nonlinear cubic splines, because preliminary inspection revealed a nonlinear relationship between this variable and home range size.

We note that, although for simplicity we refer to our spatial estimates as home ranges, they should be considered a spatial proxy, such as a multiyear activity area or “area of influence,” rather than true home range delineations.

#### P2 and P3: intrasexual territoriality

We used a resource selection function (RSF) approach (Boyce et al. 2002; Manly 2002) to test for spatiotemporal patterns in the noninvasive genetic sampling data indicating territorial exclusion. The rationale behind this application of RSF is based on two premises: (1) territoriality can be viewed as a continuum along a gradient of exclusiveness (Maher and Lott 1995) where the geographic space of one animal's territory is at least in principal available to other conspecifics in its neighborhood and (2) neighbors are less likely to be located within that territory than would be predicted by chance.

A list of individuals with ≥5 relocations and a known date of death was compiled (“focal individuals”). We used this comparatively low threshold for the required number of relocation in order to ensure that sufficient animals were available as focal individuals for the analysis. Multiyear kernel home ranges were calculated for all focal individuals based on their noninvasive genetic sampling relocations using R function “kernelUD”.

**Figure 2:**
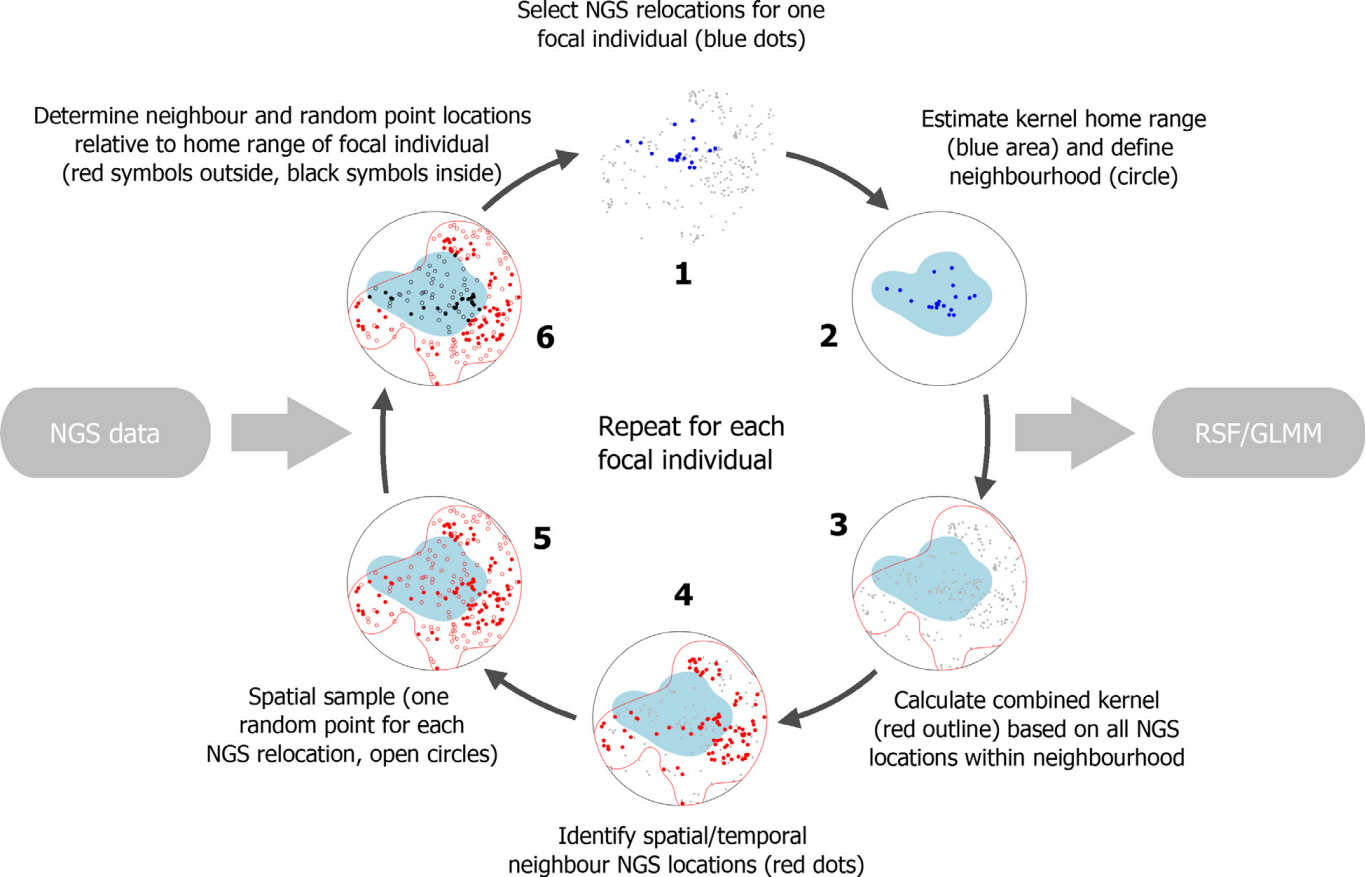
Schematic of the main steps involved in data preparation for resource selection function (RSF) analysis to test for evidence of territoriality using wolverine noninvasive genetic sampling data.

Due to the comparatively small number of samples for most individuals, kernel home ranges were sensitive to extreme spatial outliers. To avoid constructing severely inflated home ranges, we excluded spatial outliers and years where an apparent shift occurred in individual home ranges over years. The identification of outliers and home range shifts was done based on visual inspection, resulting in the exclusion of 16 scat locations (of a total of 360) from 11 individuals. The removal of outliers only affected the construction of multiyear home ranges of focal animals. The subsequent analysis was based on all samples from all individuals in the neighborhood (see below). Aside from this filter, we left the data unaltered during the analysis, taking a conservative approach that presumed that patterns worth reporting should be sufficiently pronounced to be observable despite noise in the data. A circular neighborhood with a radius of 45 km was constructed around the sample location centroid of each focal individual. Other individuals that had spatial points located inside this area were designated as “neighbors”. This neighborhood radius was selected because the resulting circle fully enclosed the largest (and irregularly shaped) 95% kernel home range of a focal animal and left room for relocations from adjacent individuals. We included only observations of neighbors collected up to 3 years prior to and up to 3 years following the year of death of each focal individual. The year of death was excluded from the analysis because not all scats from neighboring individuals found during that year could with certainty be designated as having been deposited before or after death of the focal animal. A 85% kernel contour was calculated using all spatial points from individuals (focal and neighbors, entire study period) that were inside the buffer area. The resulting polygon delineated the area searched and available to wolverines, thereby excluding unsearched areas or nonhabitat without having to perform a habitat suitability analysis explicitly. For each included scat location of a neighbor individual, we generated one simulated location via random spatial sampling within the kernel. For each point (real and random), we determined whether it fell within or outside the focal individual's home range (95% kernel isocline). An 85% kernel contour was chosen for the searched and available area to obtain a comparatively tight delineation around the relocation point cloud, thereby avoiding drawing spatial samples from a wide and potentially unused or unsearched buffer. A diagram of the procedure for preparing the data for RSF analysis is shown in Figure 2.

We fit generalized linear mixed effects models with a logit link using function “glmer” (R package “lme4”; Bates et al. 2013) to the processed data. We used location of a point inside the 95% kernel home range as the response (true/false) and the following fixed effects as predictors: (1) real/random points, (2) sex of the neighbor individual (female/male), (3) sex of the focal individual (female/male), and (4) whether the given observation was made before or after the focal individual had died. The most complex model that we considered included all possible interactions between predictors. To account for nonindependence between observations from the same neighbor–focal pair over multiple years, we included both the individual ID of focal and neighbor individuals as random effects. We selected the random effects structure using likelihood ratio tests, and we used the R function “dredge” (package “MuMIn” Barton 2012) to select fixed effects, because we had no reason to exclude any particular combination of predictor terms from consideration, with the exception of the term denoting whether a point was a true scat location or a simulated one (real/random). Models with all possible combination of terms (148 candidate models), including the most complex model, were compared using AIC, from which the model with the lowest AIC value was selected as the top model. All statistical analyses were performed using R (R Development Core Team, 2014).

To evaluate the consistency of predictions under changing assumptions about what constitutes a wolverine's “area of influence,” we repeated the analysis for 75% and 50% kernel vertices for the focal animal.

## Results

### General summary

Between 2001 and 2012, 2346 fecal samples with wolverine as the suspected host were collected, of which 1389 samples with complete collection records (including date and coordinates) were successfully genotyped, representing 281 unique wolverine individuals (146 males, 135 females). Among these, 32 (17 males, 15 females) met the conditions to be included in the analysis as focal individuals (Fig. S1), and 220 individuals fell within the buffer surrounding the home ranges of one or more focal individuals and were thus included as neighbors (counting also individuals that were designated as focal individuals in other pairings).

### P1. Home range size and sex

Linear regression revealed a pronounced effect of sex on 95% kernel home range size (*β*_log(HR size)_ = 0.826, SE = 0.3221, *t* = 2.567, df = 56, *P* = 0.013). After controlling for a positive nonlinear relationship between the number of years an individual was monitored and its home range size estimate, the model‐predicted annual home range sizes of male wolverines (757 km^2^, 95% CI: 485–1180 km^2^) were on average 2.3‐times larger than those predicted for females (331 km^2^, 95% CI: 193–566 km^2^).

Even at a larger number of relocations per individual than available in our study, both kernel and minimum convex polygon (MCP) home ranges can show substantial variation with changing number of relocations in large carnivores (Arthur and Schwartz 1999). We used simulations with repeated subsampling of each wolverine's relocation data to assess the impact of sample size (number of relocations per individual) on the home range size estimate for both kernel and MCP. Consistent with the patterns reported by Arthur and Schwartz (1999), linear mixed regression (with log‐transformed home range size as the response, number of relocations as the predictor, and individual ID as random effect on the intercept) showed that whereas kernel home range estimates (back‐transformed) tended to decrease with growing number of relocations, MCP estimates increased. The rate of change in HR size was greater for MCP estimates (*β*_log(HR)˜*N*_ = 0.17, SE* *= 0.01, *t* = 19.04) than for the kernel approach (*β*_log(HR)˜*N*_ **= −0.01, SE = −0.002, *t* = −3.8), justifying preference for the latter in our study. For example, an increase of 5–10 relocations brought along an increase in 95% MCP estimates by 281 km^2^, while the same increase in number of relocations resulted in a reduction of 95% kernel by 160 km^2^.

### P2–P3. Intrasexual territoriality

**Figure 3:**
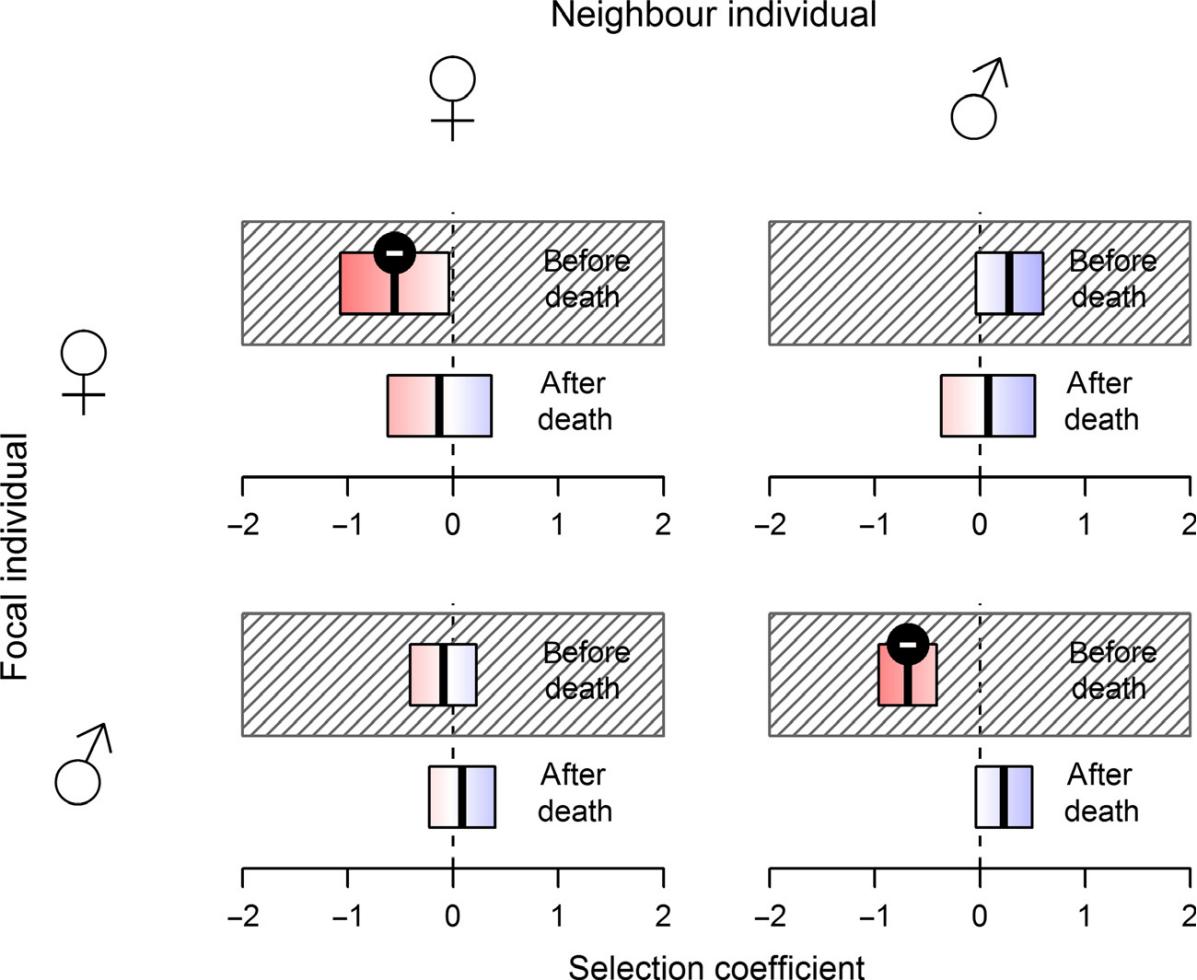
Generalized linear mixed effects model‐predicted selection coefficients for genetic sample locations of neighbor individuals within a focal individual's home range (95% kernel) before (hashed region) and after the focal individual's death. Negative coefficient values (red) indicated avoidance or exclusion, positive coefficients (blue) attraction. Predictions are shown for both same‐sex and opposite‐sex pairings. Selection coefficients significantly different from 0 are marked with the sign indicating the direction of the effect.

The most complex model (95% kernel‐delineated focal territories; real vs. random point × before vs. after death × focal individual sex × neighbor individual sex) emerged as the top model based on AICc (weight: 0.59), with a ∆AICc of 2.8 separating it from the second best model. We therefore used the saturated model for estimating coefficients and generating predictions. Selection coefficients were negative for same‐sex pairings of neighbor and focal individual prior to the focal individual's death (female–female: coef = −0.61, SE = 0.26, *z* = −2.35, *P* = 0.019; male–male: coef = −0.55, SE = 0.14, *z* = −3.95, *P* < 0.001). After death of the focal individual, selection coefficients for same‐sex pairings were no longer significantly different from 0 (female–female: coef = 0.07, SE = 0.26, *z* = −0.26, *P* = 0.793; male–male: coef = 0.17, SE = 0.13, *z* = 1.27, *P* = 0.204). For pairings of opposite sex, selection coefficients were not significantly different from 0, regardless of sex and whether sampling was conducted before or after the focal animal's death. Coefficients for pairwise interactions for each combination of the sexes are shown in Figure 3.

The model with 75% kernel multiyear home ranges showed predictions for intrasexual territoriality similar to the model with 95% kernel contours. The effect of female avoidance was no longer detectable when multiyear home ranges were delineated as 50% kernel contours, but male–male avoidance remained significant also at this level. The coefficients estimated during these alternative analyses are presented in the supplementary material (Fig. S2).

## Discussion

This study provides compelling evidence that noninvasive genetic sampling data can indeed be used to obtain qualitative and quantitative information about animal spatial behavior, specifically territorial interactions. We deliberately chose a species with a known social system for this study and found that the wolverine's sex‐specific differences in home range size and intrasexual territoriality were conspicuously manifested in the spatial configuration of noninvasive genetic sampling data. Male wolverine home ranges derived from noninvasive genetic sampling data were more than two‐times larger than those of females (P1), a pattern that had been reported previously from telemetry studies in Scandinavia (Landa et al. 1998; Persson et al. 2010) and elsewhere (Inman et al. 2012). The estimated sizes of average annual home ranges based on noninvasive genetic sampling data in our study (females 331 km^2^; males 757 km^2^) were also comparable to home range sizes reported for wolverines in Scandinavia earlier (Landa et al. 1998: females 274 km^2^; males 663 km^2^; Persson et al. 2010: females 170 km^2^; males 669 km^2^). Our analysis confirmed that individuals showed lower preference for areas used by other conspecifics of the same, but not the opposite sex (P2). Intrasexual territoriality in wolverine has been noted before: home ranges of males tend to overlap with one or more female home ranges, whereas home ranges of same‐sex adults do not or only minimally overlap each other (Persson et al. 2010; Inman et al. 2012). The apparent male–male avoidance was manifested in our analysis at all three levels (50%, 75%, and 95%) of kernel contour delineation of territories, whereas the female–female avoidance was detectable at 75% and 95% kernel contours.

No territory is held by the same individual or group indefinitely, and territory turnover has direct consequences for the spatial configuration and dynamics of populations of territorial species. Studies on turnover have focused primarily on birds, in part due to conspicuous territorial display and defense (Catchpole et al. 2003). Several mechanisms have been proposed by which territory turnover can occur, including inheritance from a group/family member, territory take‐over by challenging and eventually evicting or killing the owner, or turnover after independent death or desertion of the previous owner (Beletsky 1992). We detected evidence of territory turnover or at least the start of the process: locations of scats suggested that wolverines were more likely to select an area used by a neighboring individual of the same sex after that individual's death (P3). Although we did not assess in this study whether and how increased usage eventually translates into establishment of new territory owner(s), the boost in selection coefficients was pronounced (Fig. 3). A few studies have assessed territory turnover in raptors using noninvasive genetic sampling by collecting and genotyping shed feathers (Booms et al. 2011; Vili et al. 2013). However, in these studies, noninvasive genetic sampling was used neither to demonstrate the spatial exclusion of other individuals nor to determine the spatial extent of the territories.

Direct monitoring of individuals using telemetry can yield large amounts of data of high temporal and spatial resolution due to the possibility of frequent individual relocations. This is out of reach for studies relying on the collection of noninvasive material samples. Detailed and frequent observations are beneficial for exploring animal interactions, including territoriality, but noninvasive genetic sampling brings other advantages. It does not require the capture, chemical immobilization, handling, and marking of animals. As a consequence, noninvasive genetic sampling does not incur the animal welfare detriments (Arnemo et al. 2006), resource drain (Bischof et al. 2009), technical challenges, and disturbance of the study animals by researchers (Ibanez‐Alamo et al. 2012) associated with traditional monitoring methods. Although the number of repeated observations (sample size) for each individual is limited in comparison with telemetry applications, noninvasive genetic sampling data collections can be implemented over large spatial extents and at the population or subpopulation level (Balkenhol and Waits 2009). Working at this scale would be prohibitively expensive for most studies that require individual capture and tracking. Furthermore, regional or national noninvasive sampling data sets already collected for other purposes provide opportunity for ancillary, yet important, ecological investigations, including spatial behavior and animal interactions.

Noninvasive genetic sampling is by no means a panacea for the challenges facing studies of territoriality and animal interactions in general. The comparatively low annual sample size (recaptures per genotype) forced us to construct home ranges from multiyear noninvasive genetic sampling data, which can be problematic for transient individuals or species that show low interannual site fidelity. Inspection of the spatiotemporal patterns in wolverine noninvasive genetic sampling recaptures indicated that most wolverines tended to stay in the same general area from year to year. Davoli et al. (2013) recently showed for another carnivore that the spatial configuration of noninvasive genetic samples reflects area use. These authors estimated an 86% overlap between noninvasive genetic sampling (using hair snares) and VHF‐derived home ranges of Eurasian lynx (*Lynx lynx*). Like us, Caniglia et al. (2014) applied kernel analysis to estimate individual ranges and pack territory sizes for wolves (*Canis lupus*) from noninvasive genetic sampling data. Although no quantitative comparison with independently constructed home ranges was conducted in the wolf study, the authors employed additional sources of information (snow tracking, camera trapping, howling surveys, and direct observations) to support noninvasive genetic sampling‐derived location data (Caniglia et al. 2014).

An important limitation associated with noninvasive genetic sampling concerns detectability. Like most other field surveys of flora and fauna, noninvasive genetic sampling of wildlife suffers from imperfect detection. Imperfect detection leads to underestimation of abundance, and bias in covariate estimates are likely if detection differs between individuals or varies over space and time (Kéry and Schaub 2012). Hierarchical approaches, such as capture–recapture (CR) analysis, can account for imperfect detection and thus eliminate or minimize bias. Recently developed spatially explicit CR models (Efford and Fewster 2013; Royle et al. 2013) use the link between space use and detection to generate spatially referenced estimates of abundance. These models have the potential to incorporate territoriality and other spatially manifested interactions between individuals (Royle et al. 2013). It is probable that wolverines in our study were not detected with equal probability across individuals, time, or habitats. Nonetheless, our analytical approach makes it unlikely that the emerging patterns were artefacts of biased detection probability, because all focal individuals also served as neighbor individuals during the analysis. As a result, if, for example, a focal animal's home range was constructed based on incomplete spatial coverage of NGS sampling, the same sampling bias would also have affected the detection of neighbor individuals and the delineation of available habitat for the RSF approach. Our conclusions are further supported by the significant increase in selection coefficients after the territory owner's death. Nonetheless, spatially explicit CR models in combination with measures of search effort (as opposed to opportunistic sampling) may eventually reveal patterns and answer questions which our present study lacked the detail to address. Spatially explicit hierarchical models that account for imperfect detection of individuals may, for example, aid in the assessment of the actual process of territory turn‐over and including the spatial and temporal course of the establishment of new territory holders(s). Similarly, the ability to estimate and account for spatial heterogeneity in detection could facilitate the identification of habitat features that make up and potentially delineate territories.

## Conclusions

Our study shows that noninvasive genetic sampling can be used to identify and quantify social organization in wildlife populations, including territorial behavior. The spatiotemporal patterns associated with territoriality in wolverines were pronounced enough to be discernible despite the limited sample size and detail of scat‐based genetic sampling compared with that of telemetry studies. This has implications for wildlife monitoring because noninvasive genetic sampling, now a widespread method for monitoring carnivores and other elusive species, offers a practical alternative or at least a complement to traditional invasive monitoring approaches, which are generally too expensive to implement over large areas or across entire populations. Existing monitoring programs relying on noninvasive genetic sampling may already yield sufficient spatial information to estimate home ranges and elucidate territoriality and spatial interactions, thereby shedding light on an additional dimension of wildlife ecology. Given ongoing technical and analytical advances in noninvasive wildlife research, we expect to see more studies using noninvasive genetic sampling to investigate animal spacing behavior and interactions in the near future.

## Data Accessibility

Wolverine noninvasive genetic sampling and mortality data are available at rovbase.no.

## Conflict of Interest

None declared.

## Supporting information


**Figure S1.** Kernel home range estimates derived from scat‐based noninvasive genetic sampling for focal individuals included in the study. **Figure S2**. GLMM‐predicted selection coefficients for genetic sample locations of neighbor individuals within a focal individual's home range before and after the focal individual's death. Results are shown for alternative analyses using focal territories delineated by 75% and 50% kernel isoclines.
