# Supplementary material for: Noninvasive genetic sampling reveals intrasex territoriality in wolverines
Source: Ecol Evol. 2016 Feb 9;6(5):1527–36. doi: 10.1002/ece3.1983 (PMC4775525; doi:10.1002/ece3.1983)
Supplement: Supplementary file 1 — Figure S1. Kernel home range estimates derived from scat‐based noninvasive genetic sampling for focal individuals included in the study. Figure S2. GLMM‐predicted selection coefficients for genetic sample locations of neighbor individuals within a focal individual's home range before and after the focal individual's death. Results are shown for alternative analyses using focal territories delineated by 75% and 50% kernel isoclines. [file ECE3-6-1527-s001.docx]

Supplementary Material, Bischof et al. _________

**
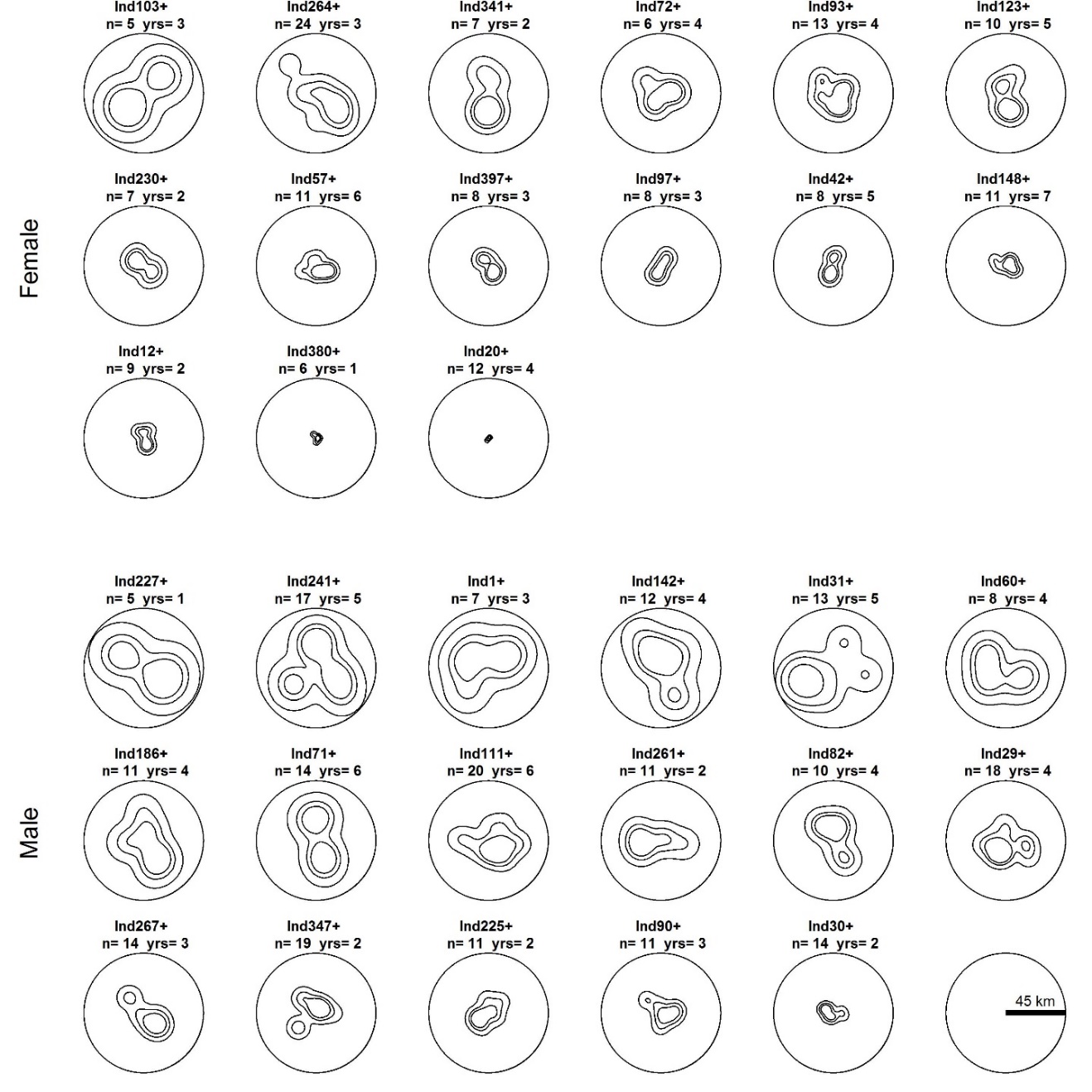
**

**Figure S1.** Kernel home range estimates (with concentric lines indicating 50%, 75%, and 95% isoclines) derived from scat-based non-invasive genetic sampling for 32 focal individuals included in the study, with circular neighbourhood area (r = 45 km). Numbers of relocations (n) and numbers of years (yrs) that were used to estimate kernel home ranges are shown above each plot.

A)

**
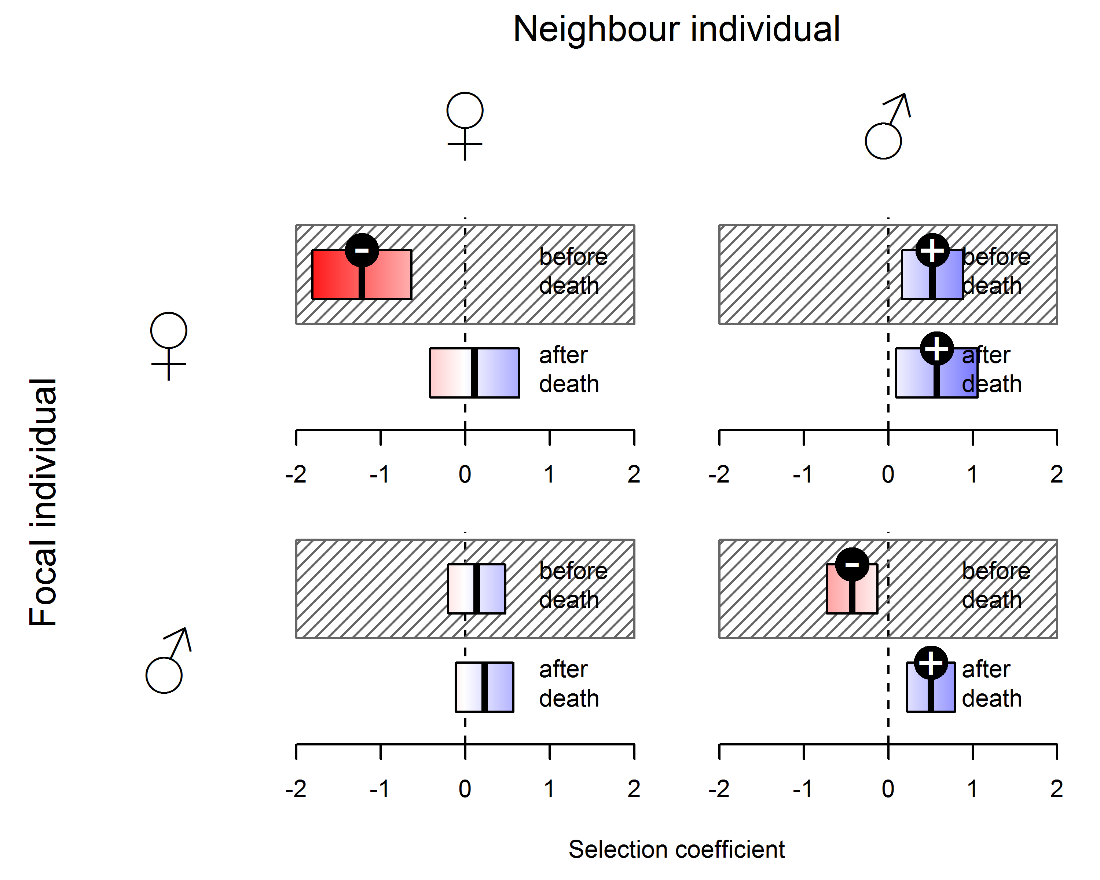
**

**B)**


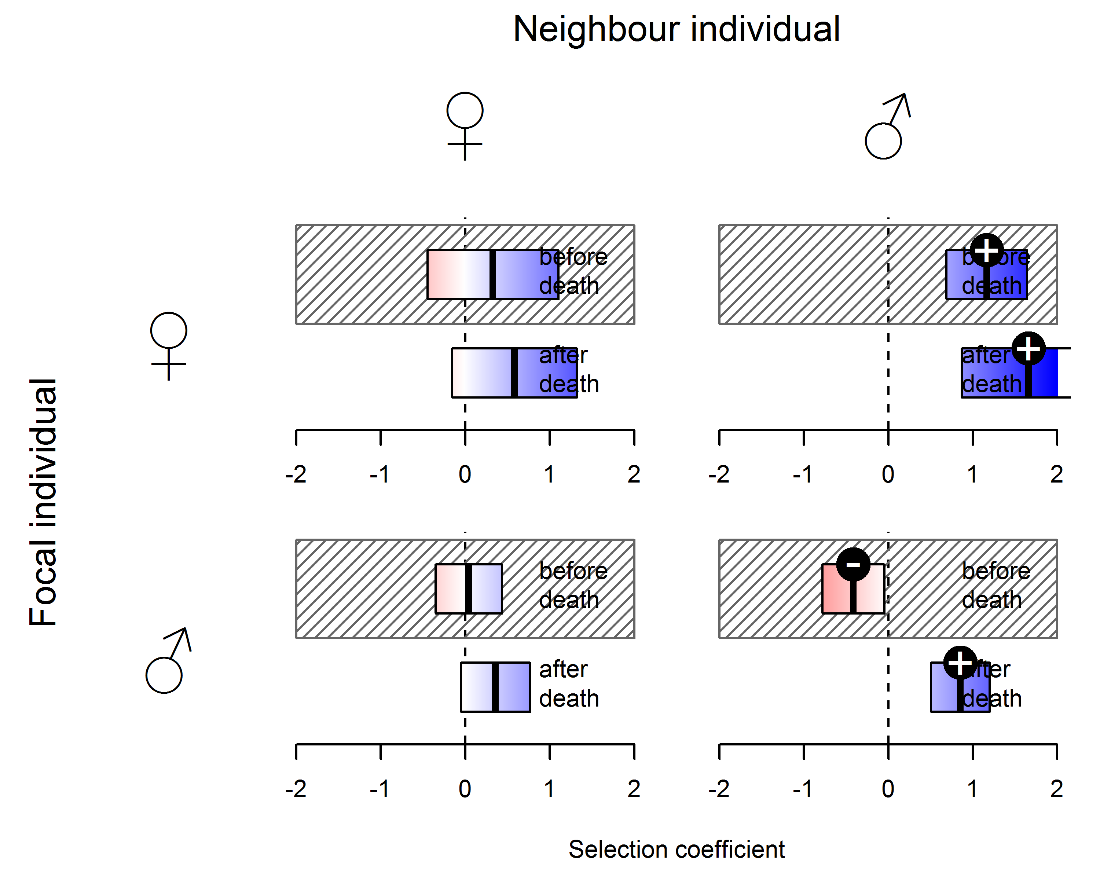


**Figure S2**. GLMM-predicted selection coefficients for genetic sample locations of neighbour individuals within a focal individual’s home range before and after the focal individual’s death. Results are shown for alternative analyses using focal territories delineated by 75% (**A**) and 50% (**B**) kernel isoclines. Negative coefficient values (red) indicated avoidance or exclusion, positive coefficients (blue) attraction. Predictions are shown for both same-sex and opposite-sex pairings. Selection coefficients significantly different from 0 are marked with the sign indicating the direction of the effect.
